# A quantitative analysis of food insecurity and other barriers associated with ART nonadherence among women in rural communities of Eswatini

**DOI:** 10.1371/journal.pone.0256277

**Published:** 2021-08-26

**Authors:** Nozipho Becker, Krishna C. Poudel, Lorraine S. Cordeiro, Aline G. Sayer, Thokozile E. Sibiya, Lindiwe N. Sibeko

**Affiliations:** 1 Department of Nutrition, University of Massachusetts, Amherst, Massachusetts, United States of America; 2 Department of Food and Nutrition Sciences, University of Eswatini, Luyengo, Kingdom of Eswatini; 3 Department of Health Promotion and Policy, University of Massachusetts, Amherst, Massachusetts, United States of America; 4 Department of Psychological and Brain Sciences, University of Massachusetts, Amherst, Massachusetts, United States of America; Baylor College of Medicine, UNITED STATES

## Abstract

**Background:**

Eswatini has the highest global prevalence of HIV despite decades of universal access to free antiretroviral therapy (ART). We conducted a mixed methods study to investigate barriers to ART adherence among women living with HIV (WLHIV) in rural communities of Eswatini. Qualitative findings were reported in our previous publication. This subsequent paper expands on our qualitative analysis to examine the magnitude to which identified barriers impacted ART adherence among WLHIV in the same communities.

**Methods:**

We used an exploratory sequential design to collect data from WLHIV (n = 166) in rural Eswatini. Quantitative data were collected using interviewer-administered survey questionnaires between October and November 2017. ART adherence was measured using the CASE Adherence Index, with scores less than 10 indicating nonadherence. Log-binomial regression models were used to examine the extent to which critical barriers affected ART adherence among study participants.

**Results:**

A majority of the women in our study (56%) were nonadherent to ART. Of the barriers identified in our prior qualitative analysis, only eight were found to be significantly associated with ART nonadherence in our quantitative analysis. These include, with adjusted risk ratios (ARR) and 95% confidence intervals (95% CI): household food insecurity (ARR: 3.16, 95% CI: 1.33–7.52), maltreatment by clinic staff (ARR: 2.67, 95% CI: 1.94–3.66), forgetfulness (ARR: 1.80, 95% CI: 1.41–2.31), stress (ARR: 1.47, 95% CI: 1.14–1.88), gossip (ARR: 1.57, 95% CI: 1.21–2.04), mode of transport (ARR: 0.59, 95% CI: 0.44–0.79), age (ARR: 0.98, 95% CI: 0.97–0.99), and lack of community support (ARR: 0.55, 95% CI: 0.35–0.85).

**Conclusions:**

Among numerous barriers identified in our study, food insecurity was found to be a significant contributor toward ART nonadherence among women living with HIV in rural Eswatini. Future strategies aimed at improving ART adherence in Eswatini should include programs which provide food and nutrition support for people living with HIV, particularly rural women living in poverty.

## Introduction

Improving quality of life for those infected with the human immunodeficiency virus (HIV) is a global ambition. Adherence to HIV treatment and retention in care of patients undergoing Antiretroviral Therapy (ART) is essential to decreasing HIV transmission and improving health outcomes for those living with HIV [1, 2]. An optimal adherence to ART is associated with increased CD4 cell counts, decreased risk of HIV transmission, sustained HIV suppression, and improved overall health [3, 4]. Poor adherence to HIV treatment is associated with reduced efficiency of viral suppression, increased risk of opportunistic infections, and increased risk of mortality [5, 6].

Effective use of ART requires a holistic approach that goes beyond just taking medication. It calls for routine monitoring of patients overall health and well-being, strategies to improve diet, increased social support, and perpetual adherence to medication for optimal viral suppression and prevention of HIV transmission [7–9]. Despite substantial progress being made regarding availability and affordability of HIV medication in Eswatini, adherence to ART continues to be a significant challenge among individuals living with HIV [10]. According to a report by the Eswatini Ministry of Health, lack of compliance to HIV treatment and care remains one of the primary challenges facing local HIV programs, and at present there is inadequate scientific data regarding ART adherence and its associated factors in the country [10].

HIV/AIDS is a major public health and socio-economic challenge for the people of Eswatini. Limited access to financial resources and difficulties in acquiring food are common concerns for many families. Approximately 69% of the people in Eswatini live below the poverty line, of which 80% reside in rural areas [11]. Current estimates indicate that 71% of Eswatini households are food insecure [12]. Over 100,000 women in Eswatini undergoing ART experience food insecurity [12, 13]. Household food insecurity (a state of inadequate access to food of sufficient quantity and quality or the inability to acquire food in socially acceptable ways) has been identified as a major barrier to ART adherence [14–16]. Prior studies examining food insecurity as it relates to HIV treatment in sub-Saharan Africa were typically conducted in urban or clinical settings, and none of these studies were conducted in Eswatini [17–19]. This study examines food insecurity specifically among women living with HIV (WLHIV) in rural Eswatini, a subgroup extremely vulnerable to poor health due to socio-economic challenges and cultural norms which traditionally oppress women [20, 21].

In 2015, Eswatini implemented a Test and Start program aimed at increasing HIV testing and ART initiation immediately after a HIV-positive diagnosis. This program greatly facilitated up scaling of ART programs nationally and consequently helped reach thousands of previously underserved people living with HIV. In 2016, the Eswatini government introduced community-cantered ART delivery models (Comm ART) intended to improve ART compliance and reduce the number of patients lost to follow-up [22]. While intervention strategies have been adopted by the Eswatini government and private organizations to help combat the spread HIV and its associated consequences, nonadherence to treatment remains a significant challenge to health facilities throughout the country. In a recent Eswatini Ministry of Health’s report that documented ART retention rates from 2008–2015, the longer patients were on treatment, a declining trend in ART retention rates was observed for those years. In 2014, ART retention rates decreased by 4% at 6 months and an additional 4% at 12 months after ART initiation among adults. Indicating that the percentage of patients remaining on treatment after ART initiation tends to decrease with an increase in duration of ART [23]. This becomes a major concern to ART programs as it suggests that patients are being lost to follow-up and likely not adhering to treatment.

For HIV programs to be successful in ensuring treatment adherence and patients’ retention to care, it is critical to investigate and identify factors that may facilitate or inhibit patients’ access to and utilization of treatment. We conducted an exploratory sequential mixed methods study investigating barriers to ART adherence among WLHIV in rural Eswatini. The initial qualitative phase was conducted among WLHIV and healthcare workers from rural communities of Eswatini between May and June 2017. Qualitative data from this study was presented in our previous publication which identified individual, household, and community level barriers to ART adherence among women in rural Eswatini [24]. Though this first aspect of the study revealed several barriers to ART adherence, due to the qualitative nature of the data, we were unable to assess the level of significance between these barriers and ART nonadherence. As part of our initial mixed methods study we intended to examine associations between household food insecurity and ART adherence using quantitative measurements. Given that lack of food, hunger, and hunger-related medication side effects were so commonly reported as major barriers to ART adherence in our qualitative analysis, we feel that there is a need to investigate in more detail the extent to which food insecurity affected ART adherence in our study. This subsequent paper expands on our qualitative analysis to examine the magnitude to which identified barriers impacted ART adherence among WLHIV in the communities we studied. More specifically, our current study aims to examine the degree of association between these barriers and ART adherence, with a specific focus on clarifying the critical association between food insecurity and ART adherence for WLHIV in rural communities of Eswatini.

## Methods

### Study design and setting

**Fig 1:**
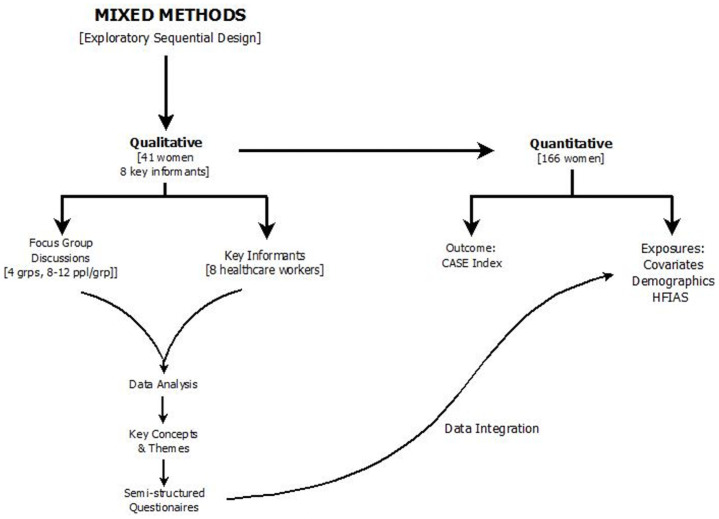
Study design.

Using a mixed methods approach, we conducted a cross-sectional study to examine barriers to ART adherence among WLHIV in communities rural of Eswatini. The study was conducted in collaboration with clinics in the Lubombo and Shiselweni regions between May and November, 2017. An exploratory sequential design (an approach utilizing two data collection phases) was used to collect data from WLHIV [25, 26]. Data were collected from four rural communities within the respective clinic’s catchment areas. In the first phase, we collected and analyzed qualitative data. Findings from the qualitative data analysis informed survey item development for the subsequent quantitative phase of the study (Fig 1). This current study reports the findings from our quantitative data analysis.

### Sampling and participants recruitment

For the quantitative phase, women were recruited using targeted community techniques with both venue recruitment (attending community outreach activities and support group meetings) and invitation notices that were posted in public locations (e.g. health facilities, community centers, constituency buildings, churches, and neighborhood care points) within the respective communities. Women who were interested were invited to participate in a survey. Women were eligible to participate in the study if they were between ages 20–49 years, and had been on ART for at least one month at the time of the surveys. Participants were compensated with a 100 Rands for travel expenses and for time taken to participate in the study.

### Sample size

Based on previous literature we estimated that approximately 50% of individuals on ART in our target communities faced one or more barriers to ART adherence. Using an alpha of 0.05 and a statistical power of 95%, 138 participants were determined to be the minimum sample required to effectively assess barriers to ART adherence in our study. With this sample size we were able to significantly detect a risk ratio of 1.3, which was comparable to prior mixed methods studies of ART adherence [27, 28].

### Data collection

Quantitative data was collected through an interviewer-administered survey questionnaire in October- November 2017. Study participants were interviewed in their native language of siSwati by trained personnel. The surveys were conducted in a central location within respective rural communities, such as local constituency buildings, churches, and neighborhood care points. The survey questionnaire included the following items: 1) Household Food Insecurity Access Scale, 2) CASE Adherence Index, and 3) a structured questionnaire including demographic information and questions on barriers to ART adherence informed by findings from our qualitative data analysis.

#### Outcome variable

ART adherence was measured using the CASE Adherence Index questionnaire, a three-item measure which assesses: (a) difficulty taking ART on time, (b) frequency of missed doses, and (c) time since the most recent missed dose [29]. Participants were asked about their ART adherence status for the month prior to the survey. They were interviewed on all three parts of the CASE Adherence Index questionnaire (S1 Appendix). Each response was assigned a score which was then used to calculate a composite value encompassing the three measures of adherence for each participant. Composite scores ranged from five to sixteen. Women who had an index score greater than 10 were classified as adherent and those with an index score less than 10 were classified as nonadherent [29]. The CASE Adherence Index questionnaire was pilot tested in Eswatini prior to conducting this study. Furthermore, previous studies of ART adherence have demonstrated the validity of this measurement instrument among individuals in African settings [30, 31].

#### Data collection of selected exposure variables

Data on household food insecurity was collected using the Household Food Insecurity Access Scale (HFIAS) [32]. Participants were asked to provide information on their household food status in the month before the survey. Women were interviewed on all three domains of the HFIAS: 1) anxiety and uncertainty about the household food supply, 2) insufficient food intake, and 3) insufficient quality of food. Food security data was used to assess two of the three HFIAS domains (i.e. insufficient food intake and insufficient quality of food) which were combined to create the food insufficiency variable. Women were classified as food insufficient if they answered “yes” to one or more of the food insufficiency questions in the second and third domains (S2 Appendix). Food insufficiency was modeled as a dichotomous variable, women that were food sufficient versus women that were food insufficient.

This food insufficiency variable was then used to create a Household Food Insecurity Index which consisted of three categories: 1) women who were from households that were food insufficient, 2) women who skipped taking medication due to hunger, and 3) those who missed doses due to hunger-related side effects from taking their medication. Next, composite scores were generated and calculated for each participant in our sample. Study participants received a composite score of 0, 1, 2, or 3 with 0 being the least food insecure and 3 the most. A composite score of 0 was assigned for women who responded “no” to all three categories, a score of 1 assigned for those who responded “yes” in only one category, a score of 2 for those who responded “yes” to two of the categories, and a score of 3 for those who responded “yes” to all three categories. Cronbach’s alpha was used to determine the reliability of all items included in the household food insecurity index, and showed a reliability coefficient of 0.80, which is regarded as acceptable [33].

As part of the survey questionnaire, participants were also asked “In the past month, have you ever skipped taking your ARVs because of any of the following reasons: hunger, hunger-related side effects, poor treatment, forgetfulness, stress, clinic too far, no money for transport, etc”. Responses to these questions were identified as barriers to ART adherence, and were modeled as dichotomous variables (e.g. hunger: yes/no). In the same questionnaire, participants were also asked to estimate travel time, transportation costs, and mode of transportation used to travel to and from the clinic or facility where they received health services. Travel time and transportation costs were modeled as continuous variables while mode of transport was modeled as a dichotomous variable, walking versus bus. Survey questionnaires were pilot tested prior to conducting this study.

### Ethical clearance

Study approval was obtained from the University of Massachusetts Amherst Institutional Review Board (Protocol ID: 2016–3309) and the Eswatini National Health Research Review Board. Permission to conduct the study was also obtained from the Eswatini Ministry of Health and participating health facilities. Written informed consent was obtained from all individual participants included in the study.

### Data analysis

Data were analyzed using STATA version 15 (College Station, TX: StataCorp LP). Data analyses were conducted at the descriptive, bivariate, and multivariable levels. Continuous variables were presented as means with standard deviations, and categorical variables were presented as frequencies and percentages. Pearson’s chi square test of significance was performed to examine associations between the outcome variable (ART adherence) and independent variables. Differences in characteristics between women who were adherent versus those who were nonadherent were assessed using the chi square test for categorical variables, and the t-test for continuous variables. The Fischer exact test was used whenever an expected cell count was less than 5. Variables that were significantly associated with ART adherence at a significance level of 25% were selected and included in multivariable regression models.

Due to high prevalence of household food insecurity in the sampled communities, we observed an insignificant variation in the household food insecurity variable, as only 1.2% of the women were from food secure households. Therefore the variable “Household Food Insecurity Index” was instead used as the main exposure variable, having wider distribution and demonstrating a better fit for the model. Multivariable log-binomial regression models were used to assess the relationship between ART adherence and Household Food Insecurity Index, while controlling for other predictors in the model. First, an initial multivariable model was fitted which consisted of all variables with a p-value less than 0.25 at the bivariate analysis. Next, we assessed the importance of each covariate based on the p-value of its Wald test. Variables that did not contribute at p<0.05 level of significance were eliminated one at a time and a new model was fitted. The likelihood ratio test was used for model comparison. Whenever the LR test p-value was greater than 0.05, variables were removed from the model. All variables that were significant at p<0.05 were kept in the final multivariable regression model.

## Results

### Demographic characteristics

This study included 166 rural women receiving ART who were surveyed between October and November, 2017 (Table 1). Participants mean age was 37 years. On average, women travelled approximately 2 hours for clinic visits, with some travelling over 8 hours (range: 0.2–8.1 hours). A majority (66.3%) of the women reported walking as their main form of travel to and from the clinic, while others mostly used public transportation (bus/kombi). More than half (54.8%) of the women were unemployed. Of the 166 women, 51.2% occasionally obtained food and/ or financial support from family members, while 10.4% occasionally received help from neighbors, friends, and community members.

**Table 1:** Demographic characteristics of study participants.

Demographic Characteristics	N = 166^a^	Percentage	Mean (SD)
Age	166		37 years (7.3)
Travel time to clinic	149		1.9 hours (1.7)
**Educational level**			
No school	39	23.5	
Primary	82	49.4	
Secondary/High	45	27.1	
**Marital Status**			
Married/ living with partner	94	56.6	
Not married	72	43.4	
**Marriage type**			
Monogamous	73	45.9	
Polygamous	25	15.7	
Committed	61	38.4	
**Employment Status**			
Not employed	91	54.8	
Employed	24	14.4	
**Income**			
< = R500	91	55.1	
R500+	74	44.9	
**Transport mode**			
Walking	110	66.3	
Public transport	56	33.7	
**Family support**			
No	80	48.8	
Yes	84	51.2	
**Community support**			
No	146	89.6	
Yes	17	10.4	

^a^ Sample sizes for variables may not add up the total due to missing data.

### Bivariate analysis

Table 2 presents measures of association between ART adherence and independent variables. We found significant associations between ART nonadherence and household food insecurity, with nonadherence being significantly higher among women who were from households that experienced food insecurity the most (74.4%, p = 0.000). In addition, ART nonadherence was significantly higher among women who reported the following barriers: clinic being too far (81.8%, p = 0.007), lack of money for transportation (76.9%, p = 0.019), forgetfulness (75.7%, p = 0.006), stress at work/home (78.6%, p = 0.001), being gossiped about (70%, p = 0.013), and experiences of maltreatment by clinic staff (79.3%, p = 0.005). Although not statistically significant, ART nonadherence was higher among women who walked to and from the clinic compared to those who travelled by public transport (bus/kombi) (p = 0.076).

**Table 2:** Results of the bivariate analysis of barriers associated with nonadherence to ART among WLHIV in rural communities of Eswatini.

Variables	Adherence N = 73		Nonadherence N = 93		P-value
**Continuous**	**Mean (SD)**		**Mean (SD)**		
Age (years)	39.4 (7.09)		37.1 (6.10)		0.000^c^*
Travel time (hours)	4.20 (1.03)		4.40 (0.84)		0.188^c^
Transportation costs (Rands)	9.56 (13.67)		8.52 (19.44)		0.201^c^
**Categorical**	**N**	**%**	**N**	**%**	
**Educational level**					0.307^a^
No school	21	53.9	18	46.2	
Primary	32	39.0	50	61.0	
Secondary/High	20	44.4	25	55.6	
**Marital Status**					0.461^a^
Married/ living with partner	39	41.5	55	58.5	
Not married	34	47.2	38	52.8	
**Employment Status**					0.758^a^
Not employed	41	45.1	50	55.0	
Employed	32	42.7	43	57.3	
**Household income**					0.691^a^
< = R500	39	42.9	52	57.1	
R500+	34	46.0	40	54.1	
**Transport mode**					0.076 ^a^
Walking	43	39.1	67	60.9	
Public Transport	30	53.6	26	46.4	
**Prescription refill**					0.036 ^a^*
Local clinic	62	41.3	88	58.7	
Other clinic	11	68.8	5	31.3	
**Alcohol use**					0.101^a^
None drinkers	61	46.9	69	53.1	
Drinkers	11	15.3	24	68.6	
**Food insecurity index**					0.000 ^a^*
0 (least food insecure)	13	76.5	5	25.5	
1	18	66.7	9	33.3	
2	21	51.2	20	48.8	
3 (most food insecure)	20	25.6	58	74.4	
**Medication side effects**					0.385^b^
No	68	44.4	85	55.6	
Yes	3	33.3	6	66.7	
**Clinic too far**					0.007^b^*
No	69	47.9	75	52.1	
Yes	4	18.2	18	81.8	
**No money**					0.019 ^a^*
No	67	47.9	73	52.1	
Yes	6	23.1	20	76.9	
**Pills too loud**					0.107^b^
No	72	45.3	87	54.7	
Yes	1	14.3	6	85.7	
**Forgetfulness**					0.006 ^a^*
No	64	49.6	65	50.4	
Yes	9	24.3	28	75.7	
**Stress**					0.001 ^a^*
No	64	51.6	60	48.4	
Yes	9	21.4	33	78.6	
**Poor treatment**					0.005 ^a^*
No	67	48.9	70	51.1	
Yes	6	20.7	23	79.3	
**Long lines at clinic**					0.172^b^
No	72	45.0	88	55.0	
Yes	1	16.7	5	83.3	
**Too many pills**					0.005^b^*
No	72	47.1	81	52.9	
Yes	1	7.7	12	92.3	
**Experience stigma**					0.078^a^
No	64	45.7	76	54.3	
Yes	6	26.1	17	73.9	
**Gossiped about**					0.013^a^*
No	51	50.0	51	50.0	
Yes	18	30.0	42	70.0	
**Feeling avoided**					0.434^a^
No	57	44.5	71	55.5	
Yes	13	37.1	22	62.9	
**Family support**					0.289^a^
No	38	47.5	42	52.5	
Yes	33	39.3	51	60.7	
**Community support**					0.162^a^
No	60	41.1	86	58.9	
Yes	70	42.9	93	57.0	

^a^: Pearson’s chi-square test;

^b^: Fisher’s exact test;

^c^: t-test;

*p<0.05.

### Multivariable analysis

Table 3 presents adjusted risk ratio (ARR) of barriers associated with ART adherence. We found significant associations between food insecurity and ART nonadherence, with women who scored the highest on the household food insecurity index being more than three times as likely to be nonadherent to ART compared to those who scored the lowest (ARR: 3.16, 95% CI: 1.33–7.52). Women who experienced maltreatment or being yelled at by clinic staff were more than twice as likely to be nonadherent to ART compared to those who did not report such treatment (ARR: 2.67, 95% CI: 1.94–3.66). Women who reported forgetting to take their medication on time and/or dates of clinic visits were almost twice as likely to be nonadherent to ART as those who did not forget (ARR: 1.80, 95% CI: 1.41–2.31).

**Table 3:** Results of multivariable analysis of barriers associated with nonadherence to ART among WLHIV in rural communities of Eswatini.

Barriers to ART Adherence	z-value	Adjusted Risk Ratio	95% CI
Age (years)	-2.77*	0.98	(0.97–0.99)
Food insecurity index			
0 (least food insecure)	reference	1.00	(-)
1	0.68	1.42	(0.52–3.88)
2	1.57	2.07	(0.83–5.16)
3 (most food insecure)	2.60*	3.16	(1.33–7.52)
Transport mode (walking vs. public transport)	-3.55*	0.59	(0.44–0.79)
Maltreatment (no vs. yes)	6.09*	2.67	(1.94–3.66)
Forgetfulness (no vs. yes)	4.09*	1.80	(1.41–2.31)
Stress (no vs. yes)	3.01*	1.47	(1.14–1.88)
Gossip (no vs. yes)	3.36*	1.57	(1.21–2.04)
Community support (no vs. yes)	-2.69*	0.55	(0.35–0.85)

z-value: test statistic; CI: confidence interval;

*p<0.05.

Similarly, women who experienced stress at work or at home (ARR: 1.47, 95% CI: 1.14–1.88), and those who felt like they were the subject of gossip by relatives, community members, and people in their social network (ARR: 1.57, 95% CI: 1.21–2.04) were more likely to be nonadherent to ART compared to those who did not experience such feelings. Women who travelled by public transport to and from the clinic were less likely to be nonadherent to ART compared to those who walked (ARR: 0.59, 95% CI: 0.44–0.79). Likewise, women who felt supported by community members were 45% less likely to be nonadherent to ART as those who did not feel supported by their communities (ARR: 0.98, 95% CI: 0.97–0.99). We found significant associations between age and ART nonadherence (ARR = 0.98, 95% CI: 0.97–0.99), with younger women being more likely to be nonadherent to ART than older women.

## Discussion

This study reports on findings from the second phase of our mixed methods study investigating barriers to ART adherence among women living with HIV in rural communities of Eswatini [24]. This current study builds on our previous qualitative analysis and reports findings from our quantitative analysis, which examined the degree to which those previously identified barriers impacted ART adherence in our target population. Of the twenty-two barriers identified in our qualitative analysis [24], only eight (i.e. household food insecurity, feelings of stress, forgetfulness, mode of transport, age, gossip, maltreatment by clinic staff, and community support) were found to be significantly associated with ART nonadherence in our quantitative analysis. More than half of the women in our study were nonadherent to ART.

The nonadherence level observed in our study was higher than rates reported in other studies of ART adherence in southern Africa [34–36], but comparable to levels that were reported in a recent study conducted in Eswatini, where 50% of study patients were found to be nonadherent to ART [37]. While studies outside of Eswatini may have reported lower rates of ART nonadherence than our study, most of those studies to have had methodological limitations [19, 34–36]. In two of the four reviewed studies, the assessment method used to measure ART adherence was neither discussed nor validated, which may have led to misclassification and /or underestimation of the reported adherence rates [34, 35]. All of the reviewed studies were conducted in urban settings, and none of which focused on rural women living with HIV, a sub-group known to be at a greater risk due to societal and socioeconomic challenges regarding access to and utilization of health services [36, 38, 39]. Additionally, adherence rates observed in previous studies may have been limited in scope, as data were collected in health facilities or clinics during follow-up visits [19, 34–36]. Individuals recruited during routine clinic visits would typically have been more proactive in monitoring their health and therefore more likely to adhere to treatment in general.

Collectively, our study findings indicate that hunger and household food insecurity were significant contributors of ART nonadherence [24]. With 98.8% of patients classified as living in food insecure households in our study population, and 72.7% of the women having experienced hunger, these data suggest that hunger and household food insecurity are common problems among people living with HIV in rural Eswatini. Nonadherence within our study population seems to occur through three mechanisms, 1) concern over lack of food or food supply within the household, 2) avoidance of medication or delaying ART when there was insufficient food in the household, and 3) skipping doses altogether when women had no prospects of procuring food due to financial constraints and/or inability to obtain food from neighbors [24]. Other studies in the region report similar findings [40–42], where lack of food and hunger have been found to be significant barriers to ART adherence among HIV-positive individuals [43, 44].

Facing extreme poverty and hunger on a daily basis while trying to cope with the complex health issues associated with HIV could understandably increase stress levels. Studies indicate that when people are stressed they are more likely to drink, and consequently, forget to take their medication [45, 46]. Our study revealed forgetfulness to be a significant barrier to ART adherence. Although our quantitative analysis did not find alcohol use itself to be significantly associated with ART adherence, forgetfulness and stress (factors associated with alcohol use) both were. This is consistent with findings from other studies investigating barriers to ART adherence among countries in southern Africa [9, 43, 47]. Our study confirms the need to intensify strategies aimed at identifying and addressing the root causes of stress for people living with HIV.

Our previous qualitative analysis revealed that transportation costs were a major barrier to ART adherence [24], however this association was not significant in quantitative analysis. In contrast, mode of transport was found to be a significant barrier to ART adherence. For rural Swazis, it is uncommon to own a car, and most of our study participants reported having to walk or use public transport to the clinic for medication refills or appointments. In our qualitative study, travel difficulties (walking long distances and lack of money for transport during clinic visits) led to patients arriving late or missing appointments, which then often led to punishment and maltreatment by clinic staff [24]. Similar findings were reported in a study conducted in Zambia, where patients reported extensive walk times, particularly when traveling in harsh weather conditions, as a main reason for missing clinic visits [40]. Similar findings have been reported from other countries in the region, where long distance travel has been shown to be a significant barrier to ART adherence [40, 46, 48]. Increased distance to one’s health facility has been associated with poor health care access and negative health outcomes [49].

Several women reported being openly reprimanded by clinic staff for missing appointments, and maltreatment by healthcare workers was found to be significantly associated with ART nonadherence in our quantitative analysis. In a recent study conducted among care givers of HIV-positive children in Eswatini, participants reported missing or skipping clinic appointments due to fear of getting yelled at and punished by healthcare workers for previously missed clinic visits [50]. Among reports from other studies in the region, maltreatment of patients has been associated with poor health care attendance and has been shown to negatively impact ART adherence [9, 51, 52].

Parallel to our qualitative findings, we found significant associations between ART nonadherence and gossip. Women who experienced being the targets of gossip (in their families, communities, and social networks) were more likely to be nonadherent to treatment. The concern women had regarding their HIV status being “discovered and gossiped about” often created barriers to the access and utilization of local ART services, and had a negative impact on ART adherence. In a qualitative study conducted among mothers and female caregivers of HIV-positive children in Eswatini, concerns over gossip were reported as one of the main reasons women avoided initiating ART treatment [48]. To our knowledge this is the first mixed methods study to report the negative effect of anxiety regarding gossip on ART adherence among WLHIV in rural Eswatini.

### Strengths

Using a mixed methods approach, we were able to identify and examine key barriers associated with ART adherence among rural Swazi women, providing us with an in-depth and comprehensive understanding of how these barriers interact and influence ART adherence. While a majority of ART adherence studies are conducted in clinical settings, our study took place within the study participant’s own communities, at familiar meeting structures with an aim to provide participants a comfortable environment in which to discuss sensitive and personal information, without fear of judgment. The sequential exploratory design of our study provided a powerful approach to identify potential mechanisms to ART adherence and allowed us to examine the extent to which critical barriers affected ART adherence. Collectively, our study findings pinpoint priority areas to target for development and implementation of future interventions aimed at increasing ART adherence in Eswatini.

### Limitations

Temporality is one of the major biases in cross-sectional studies. Because the exposures and outcomes were assessed simultaneously, causality cannot be ascertained from findings of this study. However, the findings of our study are comparable to other cross-sectional studies that have been conducted in sub-Saharan Africa on barriers to ART adherence. This study relies on self-reported adherence rates which may be susceptible to human error and may be influenced by recall bias. While data obtained using the CASE adherence index questionnaire was self-reported, this measurement instrument has been validated in prior studies and has been previously used to examine ART adherence and associated factors among individuals in African settings [29–31]. In this cross-sectional study, selection bias may have occurred via differential participation. Since participants were recruited using targeted structures in the respective communities, it is possible that participants who were concerned with stigma or accidental disclosure of HIV status, those who were too sick to respond to invitation notices, or those who could not attend community outreach activities may not have participated in the study. This may have biased the results toward the null and led to an underestimation of relative risk. Given the sensitivity of the subject matter, the findings may also have been affected by social desirability bias. In this case, participants could have intentionally underreported their food insecurity and/or nonadherence status in order to impress the research staff and/or fellow community members during the interview process. This may have resulted in misclassification and would have led to an underestimation of the true association between ART adherence and food insecurity. However, we believe the impact from social desirability bias would have been minimal as interviews were conducted in private at neutral locations within the respective communities. In addition, participants were interviewed in their language of siSwati (helping them feel less intimidated) and were constantly encouraged to provide responses that were truthful, honest, and accurate during data collection. The ability to generalize these findings may be limited to women receiving ART in rural communities where medication is provided for free in health facilities. The behavioral mechanisms linking hunger, food insecurity, and ART may vary with regard to availability and access to antiretroviral therapy. The study could not be generalized to women in urban settings, women below 20 years of age, as well as those in developed countries, or those from countries where HIV medications are not provided for free.

## Conclusions

The impact of food insecurity and hunger on ART adherence is a persistent and pervasive challenge. Providing food supplements and nutritional support have been shown to improve ART adherence [8]. We believe that future strategies aimed at improving ART adherence and ending AIDS in Eswatini should include programs which provide food and nutrition support for people living with HIV, particularly rural women living in poverty.

## Supporting information

S1 Appendix CASE adherence index questionnaire. (PDF)

S2 Appendix Household Food Insecurity Access Scale (HFIAS). (PDF)
