# Supplementary material for: A quantitative analysis of food insecurity and other barriers associated with ART nonadherence among women in rural communities of Eswatini
Source: PLoS One. 2021 Aug 26;16(8):e0256277. doi: 10.1371/journal.pone.0256277 (PMC8389413; doi:10.1371/journal.pone.0256277)
Supplement: S1 Appendix — (PDF) [file pone.0256277.s001.pdf]

S1 Appendix. CASE Adherence Index Questionnaire

Case Adherence Index questionnaire

A1. How often do you feel that you have difficulty taking your HIV medications on time? By 'on time' we mean no more than two hours before or two hours after the time your doctor told you to take it.
[4] Never
[3] Rarely
[2] Most of the time
[1] All of the time

A2. On average, how many days per week would you say that you missed at least one dose of your HIV medications?
[1] Everyday
[2] 4–6 days/week
[3] 2–3 days/week
[4] Once a week
[5] Less than once a week
[6] Never

A3. When was the last time you missed at least one dose of you HIV medications?
[1] Within the past week
[2] 1–2 weeks ago
[3] 3–4 weeks ago
[4] Between 1 and 3 months ago
[5] More than 3 months ago
[6] Never

INDEX SCORE: _________

>10 = good adherence
≤ 10 = poor adherence

Mannheimer S, Mukherjee R, Hirschhorn L, Dougherty J, Celano S, Ciccarone D, et al. The CASE adherence index: A novel method for measuring adherence to antiretroviral therapy. AIDS Care. 2006;18(7):853–61.
