# Supplementary material for: A quantitative analysis of food insecurity and other barriers associated with ART nonadherence among women in rural communities of Eswatini
Source: PLoS One. 2021 Aug 26;16(8):e0256277. doi: 10.1371/journal.pone.0256277 (PMC8389413; doi:10.1371/journal.pone.0256277)
Supplement: S2 Appendix — (PDF) [file pone.0256277.s002.pdf]

S2 Appendix. Household Food Insecurity Access Scale (HFIAS)

No.	Question	Response Options
**1st Domain: Anxiety and uncertainty about the household food supply**		
1.	In the past four weeks, did you worry that your household would not have enough food?	0 = No (skip to Q2)<br>1=Yes
1.a	How often did this happen?	1 = Rarely<br>2 = Sometimes<br>3 = Often
**2nd Domain: Insufficient quality of food**		
2.	In the past four weeks, were you or any household member not able to eat the kinds of foods you preferred because of a lack of resources?	0 = No (skip to Q3)<br>1=Yes
2.a	How often did this happen?	1 = Rarely<br>2 = Sometimes<br>3 = Often
3.	In the past four weeks, did you or any household member have to eat a limited variety of foods due to a lack of resources?	0 = No (skip to Q4)<br>1 = Yes
3.a	How often did this happen?	1 = Rarely<br>2 = Sometimes<br>3 = Often
4.	In the past four weeks, did you or any household member have to eat some foods that you really did not want to eat because of a lack of resources to obtain other types of food?	0 = No (skip to Q5)<br>1 = Yes
4.a	How often did this happen?	1 = Rarely<br>2 = Sometimes<br>3 = Often
**3rd Domain: Insufficient food intake**		
5.	In the past four weeks, did you or any household member have to eat a smaller meal than you felt you needed because there was not enough food?	0 = No (skip to Q6)<br>1 = Yes
5.a	How often did this happen?	1 = Rarely<br>2 = Sometimes<br>3 = Often
6.	In the past four weeks, did you or any other household member have to eat fewer meals in a day because there was not enough food?	0 = No (skip to Q7)<br>1 = Yes
6.a	How often did this happen?	1 = Rarely<br>2 = Sometimes<br>3 = Often
7.	In the past four weeks, was there ever no food to eat of any kind in your household because of lack of resources to get food?	0 = No (skip to Q8)<br>1 = Yes
7.a	How often did this happen?	1 = Rarely<br>2 = Sometimes<br>3 = Often
8.	In the past four weeks, did you or any household member go to sleep at night hungry because there was not enough food?	0 = No (skip to Q9)<br>1 = Yes
8.a	How often did this happen?	1 = Rarely<br>2 = Sometimes<br>3 = Often

9.	In the past four weeks, did you or any household member go a whole day and night without eating anything because there was not enough food?	0 = No (questionnaire is finished)<br>1 = Yes
9.a	How often did this happen?	1 = Rarely<br>2 = Sometimes<br>3 = Often

Coates J, Swindale A & Bilinsky P. Household Food Insecurity Access Scale (HFIAS) for Measurement of Household Food Access: Indicator Guide. Available from: https://www.fantaproject.org/sites/default/files/resources/HFIAS_ENG_v3_Aug07.pdf. Accessed June 12, 2014.
